# Supplementary material for: Administration of an Acidic Sphingomyelinase (ASMase) Inhibitor, Imipramine, Reduces Hypoglycemia-Induced Hippocampal Neuronal Death
Source: Cells. 2022 Feb 14;11(4):667. doi: 10.3390/cells11040667 (PMC8869983; doi:10.3390/cells11040667)
Supplement: Supplementary file 1 [file cells-11-00667-s001.zip › cells-1510067-supplementary.pdf]

Supplementary Figure S1.

pmol/mg protein

	C14	C16	C18:1	C18	C20:1	C20	C22	C24:1	C24	C26:1	C26
Sham-Veh_1	6.01	45.06	19.60	707.19	9.01	69.65	46.44	416.89	134.00	38.41	22.38
Sham-Veh_2	6.27	35.62	17.32	811.30	7.55	45.92	30.14	327.12	92.60	32.18	16.81
Sham-Veh_3	6.57	53.15	23.03	748.05	10.94	81.22	60.79	548.64	178.37	48.92	23.52
Ave.	6.29	44.61	19.98	755.51	9.17	65.60	45.79	430.88	134.99	39.84	20.90
S.D.	0.28	8.77	2.88	52.45	1.70	18.00	15.34	111.42	42.89	8.46	3.59
S.E.	0.16	5.06	1.65	30.28	0.98	10.38	8.85	64.32	24.7	4.88	2.07
Sham-IMP_1	4.98	35.90	18.51	649.34	9.32	63.58	45.70	407.16	127.84	38.76	19.54
Sham-IMP_2	4.95	38.98	16.16	680.76	7.69	59.49	48.02	357.73	121.02	34.70	25.40
Sham-IMP_3	5.10	42.49	17.70	574.38	7.85	58.38	46.58	368.06	133.59	33.70	21.89
Ave.	5.01	39.12	17.46	634.82	8.29	60.48	46.77	377.65	127.48	35.72	22.27
S.D.	0.08	3.30	1.20	54.66	0.90	2.74	1.17	26.07	6.30	2.68	2.95
S.E.	0.05	1.90	0.68	31.55	0.51	1.58	0.67	15.05	3.63	1.54	1.70
HG-Veh_1	8.38	62.25	24.94	858.74	10.29	80.89	62.12	567.91	190.63	52.28	26.02
HG-Veh_2	8.94	65.02	24.54	990.03	10.31	90.86	67.26	587.67	211.86	53.36	26.65
HG-Veh_3	8.39	64.92	22.78	853.24	11.40	84.33	57.39	552.30	200.97	54.56	29.64
HG-Veh_4	7.62	84.12	28.85	883.07	10.27	86.57	85.18	583.17	203.04	51.20	22.46
HG-Veh_5	8.62	65.46	23.76	890.30	10.83	91.31	62.79	675.29	198.26	44.35	28.29
HG-Veh_6	10.51	77.55	25.71	919.50	12.41	79.25	75.21	498.52	231.51	61.85	34.15
Ave.	8.74	69.88	25.09	899.15	10.91	85.53	68.32	577.48	206.04	52.93	27.86
S.D.	0.96	8.80	2.09	50.49	0.85	5.00	10.21	57.72	14.24	5.64	3.92
S.E.	0.39	3.59	0.85	20.61	0.34	2.04	4.16	23.56	5.81	2.30	1.60
HG-IMP_1	6.35	45.68	19.41	660.87	7.78	58.83	43.47	378.16	133.22	34.88	20.03
HG-IMP_2	8.47	64.78	23.23	815.31	9.88	81.52	60.74	475.39	181.54	44.17	27.51
HG-IMP_3	5.41	40.42	18.81	621.26	11.09	64.95	51.05	457.24	155.59	46.95	28.41
HG-IMP_4	6.65	48.82	19.10	543.43	9.89	70.28	59.51	433.08	179.83	49.48	44.56
HG-IMP_5	7.27	52.55	23.31	645.10	9.70	50.65	62.40	530.74	158.70	39.69	23.79
HG-IMP_6	5.87	44.84	16.92	527.83	10.38	88.06	45.60	429.30	199.13	48.47	22.63
Ave.	6.67	49.51	20.13	635.63	9.78	69.04	53.79	450.65	168.00	43.94	27.82
S.D.	1.08	8.50	2.58	103.28	1.10	13.98	8.20	51.16	23.42	5.66	8.76
S.E.	0.44	3.47	1.05	42.16	0.45	5.71	3.34	20.88	9.56	2.31	3.58

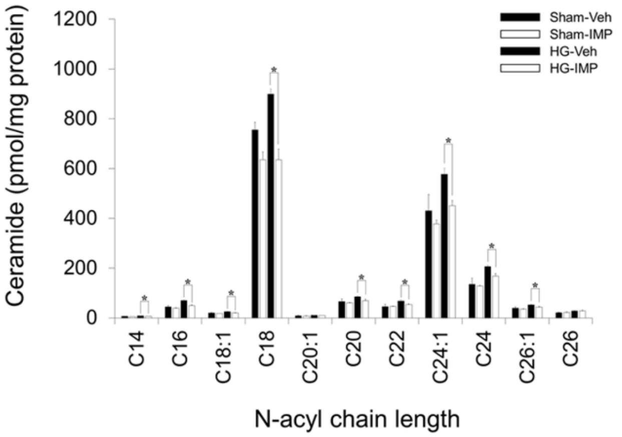

Figure S1. An analysis of 11 species of ceramide contents following N-acetyl chain length: (A) an analysis of 11 ceramide species found in common neurological diseases showed that all types of ceramide increased in the hippocampus after hypoglycemia. In particular, C18 and C24:1 remained at high concentrations under normal conditions, and both types were found to be remarkably increased in the hypoglycemia-vehicle group. On the other hand, in the imipramine-treated group after injury, all 11 ceramides were reduced and both C18 and C24:1 were significantly reduced, after having been excessively increased due to hypoglycemia. (B) The bar graph represents an analysis of 11 types of ceramide contents following N-acetyl chain length in the hippocampus. Data are mean ± S.E.M., n = 3 for each sham group. N = 6 for each hypoglycemia group. * Significantly different from the vehicle-treated group at $p < 0.05$.

Supplementary Table S1. Physiological parameters of animals

A

Glucose level (mM/L)	Before	During hypoglycemia	After
Sham-Veh	5-6	-	-
Sham-IMP	5-6	-	-
HG-Veh	3-4 (after fasting)	< 1	Recovery normal blood glucose level (5-6mmol/L)
HG-IMP	3-4 (after fasting)	< 1	Recovery normal blood glucose level (5-6mmol/L)

B

Temperature (°C)	Before	During hypoglycemia	After
Sham-Veh	36.5-37.0	-	-
Sham-IMP	36.5-37.0	-	-
HG-Veh	36.5-37.0	37.0-37.8	36.5~37.0
HG-IMP	36.5-37.0	37.0-37.8	36.5~37.0

C

Body Weight (g)	Before	During hypoglycemia	After
Sham-Veh	300-330	-	330-350
Sham-IMP	300-330	-	330-350
HG-Veh	300-330	300-330	300-330
HG-IMP	300-330	300-330	300-330

D

Heart rate (bpm)	Before	During hypoglycemia	After
Sham-Veh	330-360	-	-
Sham-IMP	330-360	-	-
HG-Veh	330-360	330-420 (bradycardia 150-210bpm for approximately 10 seconds)	330-360
HG-IMP	330-360	330-420 (bradycardia 150-210bpm for approximately 10 seconds)	330-360

E

Blood pressure (mmHg)	Before	During hypoglycemia	After
Sham-Veh	80-120	-	-
Sham-IMP	80-120	-	-
HG-Veh	80-120	150-230	80-120
HG-IMP	80-120	150-230	80-120

Table S1. Physiological parameters of animals on before hypoglycemia, during hypoglycemia. and after hypoglycemia: (A) blood glucose level (mM/L). (B) Temperature (°C). (C) Body weight (g). (D) Heart rate (bpm). (E) Blood pressure (mmHg). There was no difference between the four groups before and after hypoglycemia on blood glucose level, temperature, blood pressure, and heart rate, except for weight loss due to surgery after hypoglycemia.
